# Association of Serum Biomarkers with the Mortality of Trauma Victims in a Level -1 Trauma Care Centre of Eastern India

**DOI:** 10.30476/BEAT.2022.89155.1222

**Published:** 2022-01

**Authors:** Kasturi Mukherjee, Debojyoti Bhattacharjee, Jayati Roy Choudhury, Raghunath Bhattacharyya

**Affiliations:** 1 *Department of Biochemistry, IPGME&R and SSKM Hospital, West Bengal, India*; 2 *Department of Obstetrics and Gynaecology, College of Medicine and Sagore Dutta Medical College, West Bengal, India*

**Keywords:** Biochemical parameters, Injury Severity Score (ISS), Mortality, Trauma

## Abstract

**Objective:**

To determine correlation of important biochemical laboratory investigations in different trauma patients and their degree of injury severity and overall mortality association.

**Methods:**

In this hospital based retrospective observational study, 238 trauma patients were divided into two groups. Group I with injury severity score (ISS)<16 and group II with ISS>16. Haemoglobin (Hb), international normalized ratio, serum creatinine, blood urea nitogen (BUN), serum electrolyte, serum uric acid and liver function parameters were recorded and statistically analyzed.

**Results:**

Group II had statistically significant (*p*<0.0001) elevated levels for referral pulse rate, creatinine, BUN, liver enzymes and decreased level in Hb% and potassium level compared to Group I. Strong positive correlation only exists between BUN and severity score, moderate positive correlation exists between creatinine, aspartate transaminase, and alanine transaminase, alkaline phosphatase and severity score and negative correlation between potassium and severity score. In this study, higher odds of high BUN and creatinine and lower potassium to normal values are associated with bad outcome such as higher mortality in the population of high ISS (>16).

**Conclusion:**

The study establishes the absolute need of doing three laboratory parameters (serum creatinine, serum blood urea nitrogen and serum potassium) instead of doing laboratory tests battery at the time of trauma victims admission and predicting survival among injured patients in trauma population from Indian settings.

## Introduction

Trauma is one of the leading causes of mortality and morbidity worldwide. The assessment of traumatic injury serum markers still remains less than optimal. It requires appropriate skill to predict the severity of injuries among trauma patients for better management outcome. The clinical status of early assessment in severely injured patients is a pivotal importance to guide surgical and intensive care management. Polytrauma patients are expected to have a higher risk of mortality than obtained by the summation of expected mortality owing to their individual injuries.

Several trauma scores have been developed to predict injury severity and the risk of mortality; the injury severity score (ISS) is the most commonly used. ISS assesses the combined effects of the multiple injured patients and is based on an anatomic injury severity classification. The term “polytrauma” has been frequently defined in terms of a high injury severity score (ISS). The injury severity score (ISS) is an anatomical scoring system that provides an overall score for patients with multiple injuries. Each injury is assigned an AIS score and is allocated to one of the six body regions (head, face, chest, abdomen, extremities (including pelvis), and external. It can be administered to people with sustained injury in more than one part of the body and it is determined to score every injury with the abbreviated injury scale (AIS). Only the highest AIS score is used in each body region. The three most severely injured body regions have their score squared and added together to produce the ISS score. The ISS score takes values from 0 to 75. A score of 1 represents a minor injury and a score of 75 represents a fatal injury. AIS scores of the three most injured body regions: A, the head, neck, and face; B, the thorax and abdomen, and C, the extremities (including the external pelvis). An ISS of 1-8 is considered minor, 9-15 moderate, 16-24 severe, and 25 and high very severe [1]. If an injury is assigned an AIS of 6 (un-survivable injury), the ISS score is automatically assigned to 75. The internationally accepted threshold of an ISS ≥ 16 is based on the description as being a mortality risk predictive of above 10% [2]. It is very difficult to apply the definition of the polytrauma in the clinical setting, along with certain limitations using the ISS as a decision-making tool in the clinical setting. The priority and precedence of diagnostic methods in patients with polytrauma is determined to an individualized basis rather than the guideline recommendations. An ideal marker is one that is quickly and simply measured in the serum. The World Health Organization (WHO) has defined each measure as a biochemical parameter, which allows the comparison of a potential biological system with any potential hazard. There is no sharp definition of biochemical markers used in injuries. Biochemical markers may be associated with the injury level in the set up with limited radiological resources. According to the patient, the markers are determined to the ascertained prognostic purpose and evaluated patho-physiological changes. Rapidly obtained biochemical parameter results perform efficiently as markers and are absolutely necessary to the diagnosis and follow-up of injuries in multi trauma patients. 

We designed this study to investigate the polytrauma severity of patients with polytrauma being defined by the new berlin definition, which admitted and treated for all trauma injuries at a level I trauma center with serum biomarkers. The objective of the present study is to evaluate the serum markers after polytrauma in comparing with the serum markers role in an assessment of injuries and the outcome of these markers prognostic. Serum creatinine, BUN, serum electrolyte, serum uric acid, liver function test parameters serve as the cornerstone of all interventions that provided to patients who have sustained severe traumatic injury. We hypothesized that hemoglobin percent (Hb %), international normalized ratio (standardized prothrombin time) (INR), serum creatinine, BUN, serum electrolyte, serum uric acid and liver function test parameters on arrival would be helpful to correlate the severity. We attempted to analyze and correlate the usefulness of these biochemical values with the severity and in-hospital mortality in severe trauma patients. 

## Materials and Methods

It is a hospital based retrospective observational study that performed in the Level I trauma care center of IPGMER and SSKM Hospital, Kolkata, West Bengal. Trauma care has been developed to provide 24 hours’ emergency services. All traumatized patients were initially resuscitated by emergency trauma team and scoring was evaluated based on clinical finding. The ISS is an anatomic scoring system that provides an overall score for patients with multiple injuries. Each injury is assigned an abbreviated injury scale score and is allocated to one of the following six body regions: head, face, chest, abdomen, extremities (including the pelvis), and external. Only the highest abbreviated injury scale score is used for each body region. The three most severely injured body regions have their scores squared and summed to produce the ISS score. 

In this observational study, formal sample size calculation is not performed. An ISS of 1-8 is considered minor, 9-15 moderate, 16-24 severe, and 25 and higher very severe [1]. All patients are divided into two groups: an ISS>16 and ISS<16 who were treated for severe trauma. 287 eligible patients retrospectively and consecutively were included in the study. 49 patients were excluded as per exclusion criteria. Among them, 238 patients were finally included in the study. Patients were divided into group I (n=125) with ISS<16 and group II (n=113) with ISS>16. The medical records and electronic laboratory results were reviewed and data were extracted on demographics injury mechanism based on ISS. Serological test was included in this study conducted for a period of 8 months from November 2019 to July 2020 in a blind fashion by a laboratory technologist who was unaware of the subjects’ clinical status, injury severity and ISS. All data are documented. All patients between 18-60 years of age with acute traumatic multiple injuries brought in the trauma care center within 4 to 6 hours after trauma. With the addition of at least one of five standardized physiological responses (hypotension [SBP≤90 mmHg], unconsciousness [GCS score≤8], acidosis [base excess≤−6.0], coagulopathy [partial thromboplastin time ≥40 s or international normalized ratio≥1.4], and age [≥70 years]) AIS≥3 for six body regions was determined [3]. Patients were excluded with burn injury, penetrating injury, un-survivable injury (AIS=6, ISS=75) or incomplete registered data, those who were discharged from the emergency department <18 years of age, elderly trauma cases, brought dead cases. Cases with missing values of any biochemical investigation under consideration were excluded from further analysis. Ethical clearance has been obtained from the ethics committee institution as per the ethical standards recommended by the Helsinki of 1975 and reviewed by the institutional research oversight committee. 

On arrival of trauma care facility emergency, scoring has been performed over all poly trauma cases. At the time of admission, data were collected according to standard hospital protocol that includes age, sex, place of stay, along with referral systolic blood pressure (SBP), referral pulse rate. All investigation details were documented. As soon as patient is being resuscitated in hospital and as part of routine clinical workout, venous blood samples were collected from each patient of poly trauma without any delay. Blood sample was collected randomly by standard veni puncture technique into plain plastic tubes and in EDTA vials using aseptic precautions. Complete clot formation was ensured prior to centrifugation. Serum was separated after centrifuging for 15 minutes and was analyzed for all the parameters on the same day. All biochemical parameters are performed in Transasia EM 360. Serum creatinine, blood urea nitrogen (BUN), uric acid and total bilirubin, total protein, albumin, alkaline phosphatise (ALP), alanine transaminase (ALP), aspartate transaminase (AST) are performed in spectrophotometric technique whereas serum sodium (Na^+)^ and potassium (K^+)^ are done in direct ISE integrated with Autoanalyser according to manufacturer’s instructions after proper validation. All Hb% is performed in fully automatic 23 Sysmex XN-550 cell counter, INR in Coagu Check XS. Data were analyzed using Med Calc statistical software for biomedical research version 19.6. As the variables in this trauma study showed normal distribution by goodness of fit test, two tailed student’s t test has been done to compare the parametric data sets. A *p*-value less than 0.05 were considered statistically significant. Referral pulse rate, creatinine, BUN, liver enzymes (ALT, AST, ALP), Hb% and potassium level are the biochemical variables that show statistically significant differences with severity of ISS score. Bivariate correlation analysis has been done with biochemical variables and severe ISS score. Odds Ratio (OR) for above and below the normal range considering 95% as confidence interval (CI) were estimated in variables showing considerable correlation. There would be no conflict of interest among the investigators of this study. Informed consent form has been signed by all the associates of polytrauma patients who are illiterate and their witness signature was taken. No conflict of interest lies among the researchers.

## Results

Between November 2019 to July 2020, 238 polytrauma patients were selected considering the inclusion and exclusion criteria. The final eligible study population comprised of two groups based on ISS, group I (n=125) with ISS<16 and group II (n=113) with ISS>16. Group I comprised of 97 men (77.6%) and 28 women (22.4%) (Table 1). The group II population had 102 men (90.3%) and 11 women (9.7%) (Table 1). Within 7 days of post admission irrespective of medical and surgical intervention, 18 patients (14.4) died from group I and 30 (24%) from group II (Table 1). Maximum Causal association of polytrauma cases has been associated with two wheeler accidents in both groups (Table 1). The mean age of polytrauma occurrence were 32±8.7 years (group I) and 30±7.4 years (group II), respectively; which shows statistical matching as there is no significant differences (*p*=0.0590) (Table 2).

**Table 1:** Demographic percentage between two groups

		**Group I (n=125)**	**Group II (n=113)**
Gender	Male	77.6% (97)	90.3%(102)
	Female	22.4%(28)	9.7%(11)
Mortality		14.4%(18)	24%(30)
Mechanism of Injury	Two wheeler accidents	72%(90)	69%(78)
	Car accidents	9%(11)	11%(12)
	Large vehicle accidents	6%(8)	7%(8)
	Fall	10%(12)	12%(14)
	Pedestrians	3%(4)	1%(1)

**Table 2:** Comparison the clinical and laboratory parameters among two groups

**Variables**	**Group I (mean**±**SD**^a^**) (n=125)**	**Group II (mean**±**SD**^a^**) (n=113)**	***p*** ** value**
ISS	11±0.9	30±0.8	<0.0001
Age of Occurrence (years)	32±8.7	30±7.4	=0.0590
Referral SBP (mm of Hg)	128±15.8	132±17.7	=0.066
Referral Pulse rate (/min)	86±1.7	98±0.9	<0.0001
Hb (gm/dl)	14.2±2.1	12.4±1.3	<0.0001
INR	1.10±1.3	1.38±1.1	=0.075
Creatinine mg/dl	1.4±0.3	1.8±0.6	<0.0001
BUN mg/dl	22±2.2	28±1.5	<0.0001
Sodium meq/L	136±5.8	138±9.7	=0.052
Potassium meq/L	3.2±0.2	2.4±0.7	<0.0001
ALT IU/L	50±1.3	84±0.7	<0.0001
AST IU/L	46±2.1	90±1.6	<0.0001
ALP IU/L	290±0.8	330±3.7	<0.0001
Total Bilirubin mg/dl	0.8±0.07	0.9±0.1	=1.000
Total Protein gm/dl	7.2±1.3	6.8±2.3	=0.095
Albumin gm/dl	3.4±1.6	3.1±0.8	=0.073
Uric acid mg/dl	6.8±3.8	7.6±2.9	=0.071

^a^SD: Standard Deviation

Group II had elevated levels for referral pulse rate, creatinine, BUN, liver enzymes (ALT, AST, ALP) compared to group I. The differences were statistically significant. (*p*<0.0001). There is a significant decrease in Hb % and potassium level in group II compared to group I (*p*<0.0001). The level of referral SBP (*p*=0.066), INR (*p*=0.075), Na^+ ^(*p*=0.052), total bilirubin (*p*=1.000), total protein (*p*=0.095), albumin (*p*=0.073) and uric acid (*p*=0.071) were comparable in two groups with respective *p* values of insignificant difference.

Bivariate correlation analysis has been performed between ISS, clinical and biochemical variables showing significant differences (either increase or decrease) i.e. ISS, referral pulse rate, Hb%, creatinine, BUN, potassium, ALT, AST and ALP in group II data (Table 3). Among them, weak positive correlation exists between referral pulse rate (r=0.31), weak negative correlation exists Hb % (r=-0.40) and ISS; moderate positive correlation exists between creatinine (r=0.57), AST (r=0.52), and ALT(r=0.49), ALP(r=0.61) and negative correlation between potassium (r=-0.65); Strong positive correlation only exists between BUN (r=0.79) and severity score (ISS).

**Table 3:** Pearson correlation between the biochemical parameters in group II cases

**Parameters**	**Correlation Coefficient (R value)**
BUN mg/dl vs. ISS	0.79
ALT IU/L vs. ISS	0.49
Creatinine mg/dl vs. ISS	0.57
Potassium meq/L vs. ISS	-0.65
AST IU/L vs. ISS	0.52
ALP IU/L vs. ISS	0.61
Referral pulse rate vs. ISS	0.31
Hb gm/dl vs. ISS	-0.40

The investigation results of group II (n=113) shows moderate and strong correlation with ISS (creatinine, AST, ALT, potassium, ALP, and BUN) and were categorized into normal range and above or below normal range considering 95% as confidence interval (CI) (Table 4). Considering normal range values of laboratory investigation of group II trauma patient data as reference category, odds ratio of low value potassium (OR 2.6500, CI1.1250 to 6.2423 and *p*=0.0258) to normal values in association with mortality of 60%, high value BUN (OR-12.6000, CI- 4.5147 to 35.1648, *p*<0.0001) in association of mortality of 80% and high value creatinine (OR- 2.4375, CI-0.9744 to 6.0972, *p*=0.0568) to normal values in association of mortality of 73% have been found, respectively.

**Table 4:** Biochemical laboratory investigation-wise survival in group II trauma patients (n=113).

**Parameters**	**Alive n (%)**	**Died n (%)**	**Odds ratio**	**95%CI** ^a^	***p*** ** value**
Potassium (3.5-5.1meq/L)					
Low	30(36%)	18(60%)	2.6500	1.1250 to 6.2423	=0.0258
Normal	53 (64%)	12(40%)			
ALT (<45U/L)					
High	57(69%)	19(63%)	0.7879	0.3283 to 1.8911	0.5935
Normal	26(31%)	11(37%)			
AST (<35U/L)					
High	61(73%)	17(57%)	0.4716	0.1973 to 1.1271	=0.0909
Normal	22(27%)	13(43%)			
ALP (44 to 147 IU/L)					
High	45(54%)	14(47%)	0.7389	0.3199 to 1.7068	=0.4787
Normal	38(46%)	16(53%)			
BUN (7-20 mg/dl)					
High	20(24%)	24(80%)	12.6000	4.5147 to 35.1648	<0.0001
Normal	63(76%)	6(20%)			
Creatinine (0.9-1.3 mg/dl)					
High	44(57%)	22(73%)	2.4375	0.9744 to 6.0972	=0.0568
Normal	39(43%)	8(27%)			

^a^CI: Confidence Interval

## Discussion

Without a clear definition of polytrauma, any attempt to compare the loads, interventions, and outcomes of polytrauma patients among various trauma centers is challenging [4]. The definition of polytrauma based on the number of injured body regions does not reflect the physiological course after injury, which can be very dynamic in nature and may profoundly influence outcomes. Considering that the present study compared serum biomarkers in a broad group of hospitalized polytrauma patients with ISS 16 as cut off, VanDerHeyden *et al*., [2] study showed that polytrauma patients with higher ISS presented significantly high morbidity and mortality [5, 6]. In the present study, there is no significant difference between occurrence age (*p*=0.05). Our study revealed that referral pulse rate, creatinine, BUN, liver enzymes (ALT, AST, ALP) are elevated with increase in ISS score whereas Hb % and potassium values decreased when there is increased trauma severity. All these biochemical parameters in group II samples showed significant correlation whereas BUN has found to have a remarkable correlation with ISS. For higher value of serum creatinine and BUN, the relative significant odds were 2.10 and 12.6, respectively, as compared to normal range; whereas lower value of potassium showed odds of 2.65 with normal values. Litofsky *et al*., [7] opined that initial hemoglobin and lowest hemoglobin after admission are independently associated with poor outcome. Patient’s outcomes after polytrauma are worse in patients having lower hemoglobin level. Takaaki Ookuma in their study stated that trauma patients presenting with hypokalemia (<3.5meq/L) at the time of admission is associated with an increased trend toward in-hospital mortality [8]. Our findings suggest that hypokalemia in trauma patients on admission reflects the severity of trauma. But sodium values exhibit no significant changes between two groups that corroborate with Duggan *et al*., [9] study. The present study revealed the elevation of all three liver enzymes and positive correlation with severity. Fox *et al*., [10] suggested that liver enzymes elevated after polytrauma which corroborates with our study. Several trauma studies showed ALP gets elevated in severe polytrauma cases [11-13]. Patients with abnormal pre-operative liver function are known to have a high complication and mortality rate after abdominal surgery. Other than liver enzymes, total bilirubin, total protein and albumin showed no significant difference with increase in trauma severity similar to other trauma study [9]. Coagulation profiles are measured in patients undergoing surgery to predict the chances of post-operative hemorrhage but there is little supportive evidence [14]. Our study revealed no significant changes in INR among groups. After trauma injury stress causes increase in uric acid [15], we did not get significant changes in uric acid in immediate samples; may be as the tissue repair and remodeling has not been started. Reduced renal perfusion due to hemorrhage and other causes extracellular volume depletion is common in polytrauma patients. The incidence of post-traumatic acute renal failure requiring hemodialysis treatment is 107 per 100,000 trauma center admissions. One-third of the patients have early ARF (3-5 days after trauma) while two-thirds develop late ARF, both with or without multi-organ failure [16]. This study exhibits strong positive correlation of BUN (r=0.79) and severity score (ISS) and moderate positive correlation exists between creatinine (r=0.57) and both of these parameters showed significant differences with increase in ISS score. Chinese researchers have highlighted the importance of close surveillance of renal function and stressed the value of renal hygiene in the severe TBI cases [17]. In another study done on database from “towards improved trauma care outcomes” (TITCO) from four Indian city hospitals suggested similar findings of significant need monitoring of the serum creatinine and BUN levels of trauma victims to prevent acute kidney injury (AKI) [18].

It has been observed that ISS>16 had a higher mortality and morbidity proportion [3]. As resource utilization becomes increasingly scrutinized, the question remaining is whether test battery should be recommended as a “routine protocol” across the entire spectrum of trauma patients. With limited resources, it will be wiser to categorize investigations for all polytrauma patients. Unadjusted odds considering the confidence intervals and *p* value (provided) to determine significance has been used as no other confounding factor affect the causal association of exposure lies. The odds ratio has been used to determine whether high level of ALT, AST, ALP, BUN, creatinine and low potassium is a risk factor for death (CI 95%) and to compare the magnitude of these risk factors for the outcome. In the present study, we have found higher odds of BUN (and creatinine to normal and lower potassium values to normal is associated with bad outcome i.e. higher mortality in the population to have high ISS (>16). The three parameters of serum BUN (mortality among ISS>16 --80%), creatinine (mortality among ISS>16-- 73%) and potassium (mortality among ISS>16--60%) have made a profound impact over the mortality of Trauma victims at the time of admission. Therefore, it would be better to recruit a facility of point of care testing (POCT) to do BUN, creatinine and potassium for improving the management of trauma patients. 

It has been noted that increased severity of polytrauma is associated with changes of certain biochemical parameters. Categorized ordering of laboratory testing should be done for better clinical outcomes of the trauma victims. It is pertinent to mention that optimal categorized sampling as screening can reduce the mean hospital cost per person in trauma centre and can improve the turnaround time of selected biochemical report parameters of these emergency samples. This will lead to better patient outcome and to develop clinical practice guidelines.

The major limitation of this study is its retrospective design. In this institution based study, we assessed the outcome based on a single one-time laboratory value. The health of trauma victim before injury was not taken into account as abnormal laboratory results are reported. External validation in large multi centric randomized trial is necessary to validate this categorization of biochemical parameters (serum BUN, creatinine and potassium) among polytrauma patients to include them into scoring system. Analysis of this study revealed that biochemical investigations at the time of admission are associated with the survival of trauma patient. 

## Declaration

### Ethics approval and consent to participate

Ethical clearance has been obtained from the ethics committee institution as per the ethical standards recommended by the Helsinki of 1975 and reviewed by the institutional research oversight committee. Informed consent form has been signed by all the associates of polytrauma patient’s who are illiterate and their witness signature was taken.

### Consent for publication

None.

### Conflict of interests

The authors declare that there is no conflict of interest.

### Funding

The authors have no financial disclosures to declare. This work was done at government hospital and no special fund was utilized.

### Authors’ contributions

First 3 authors have conceived the concept of the article and collected the data. The corresponding author performed the analysis and interpretation of data for the article; all the authors have made a substantial contribution to the draft of the article, revised it critically and approved the version to be published.

### Acknowledgements

We are grateful to laboratory technologists of Trauma Care centre, IPGMER & SSKM Hospital for their support.
